# Higher Anti-Liver Fibrosis Effect of *Cordyceps militaris*-Fermented Product Cultured with Deep Ocean Water via Inhibiting Proinflammatory Factors and Fibrosis-Related Factors Expressions

**DOI:** 10.3390/md15060168

**Published:** 2017-06-08

**Authors:** Yu-Ping Hung, Chun-Lin Lee

**Affiliations:** Department of Life Science, National Taitung University, 369, Section 2, University Rd., Taitung 95092, Taiwan; tomato.1127@hotmail.com

**Keywords:** liver fibrosis, deep ocean water, *Cordyceps militaris*, cordycepin, adenosine

## Abstract

Deep ocean water (DOW) has been shown to enhance the functional components of fungi, resulting in increased health benefits. Therefore, using DOW for culturing fungi can enhance the cordycepin and adenosine of *Cordyceps militaris* (CM) and its protective effects on the liver. In this study, the antiliver fibrosis effects and mechanisms of ultrapure water-cultured CM (UCM), DOW-cultured CM (DCM), synthetic water-cultured CM, DOW, cordycepin, and adenosine were compared in the liver fibrosis mice induced by intraperitoneal injections of thioacetamide (TAA). The results indicated that DCM exhibited superior performance in reducing liver collagen accumulation, mitigating liver injuries, inhibiting proinflammatory factors and fibrosis-related factor (TGF-β1, Smad2/3, α-SMA, COL1A1) expression compared with UCM. DOW, cordycepin, and adenosine also performed antiliver fibrosis effect. Therefore, because DCM is rich in DOW and functional components, it can achieve anti-liver fibrosis effects through multiple pathways. These ameliorative effects are considerably superior to those of UCM.

## 1. Introduction

The inflammatory response to chronic liver disease is generally highly correlated with liver fibrosis [1]. Such a response is induced when cytokines are produced by the hepatocytes and Kupffer cells in response to liver damage [2]. The occurrence of the cytokine-induced inflammatory response prompts the activation of hepatic stellate cells (HSCs) [3]. The activated HSCs secrete more cytokines, causing continual inflammatory responses, which subject the HSCs to a state of ongoing activation. This further triggers fibrosis and cell proliferation [1]. Among the cytokines secreted by the activated HSCs, the transforming growth factor-β1 (TGF-β1) plays a crucial role in regulating clinical and experimental liver fibrosis. TGF-β1 can indirectly stimulate the activated HSCs to produce substantial amounts of extracellular matrix (ECM) and inhibit its degradation [4]. In addition, TGF-β1 can regulate the proliferation of HSCs by transmodulating platelet-derived growth factor (PDGF) receptors [5]. It also activates HSCs through a TGF-β1/Smad pathway, causing liver fibrosis [5].

Deep ocean water (DOW) is rich in inorganic nutrients and mineral content. Numerous studies have reported that DOW has multiple health benefits, such as improving skin inflammation [6] and exhibiting hypolipidemic effects, antidiabetic effect [7], and antiobesity effects. In addition to these health benefits, DOW increases the functional components of monascin and ankaflavin in red mold dioscorea, and the concentration of triterpenoids, polysaccharides, and total flavonoids in *Antrodia camphorata* [8,9]. Furthermore, red mold dioscorea fermented with DOW exhibits stronger hypolipidemic and antiobesity effects compared with that fermented in pure water [8,10]. Finally, DOW-cultured *A. camphorata* also has stronger hepatoprotective effects compared with that cultivated with pure water [9].

*Cordyceps militaris* (CM) is an entomogenous fungus widely used in Asia as a medicinal fungus. Cordycepin is a biologically active substance unique to the genus *Cordyceps*. Previous studies have shown that CM-fermented cordycepin has antioxidative and anti-inflammatory effects [11,12]. Cordycepin, a CM metabolite, can inhibit Smad phosphorylation and phosphate levels, thereby interfering with TGF-β1 signaling and inhibiting the activation of HSCs [13]. The described studies have indicated that using DOW as the culturing water can help increase the active components of CM and its liver protection functions. Therefore, in the current study, we investigated the preventive and ameliorative effects of DOW-cultured CM (DCM) against liver fibrosis and the mechanism underlying such effects. Thioacetamide (TAA) was used to induce liver fibrosis in male BALB/c mice to investigate whether DCM can increase the hepatoprotective effects of CM and to understand its influence on serum liver function. We then conducted histological staining to assess liver cell damage and collagen accumulation. The protein expression of profibrogenic risk factors (TGF-β1 and smooth muscle α-actin (α-SMA) and inflammatory factors (interleukin 6 (IL-6), interleukin1β (IL-1β), tumor necrosis factor alpha (TNF-α), and nuclear factor-κB (NF-κB)) in liver tissue was also investigated to determine the factors and possible mechanisms of DOW’s influence on the antiliver fibrosis effect of CM. In addition, we simultaneously investigated the antiliver fibrosis effect of CM cultured with synthetic water (SW) (SCM), which is prepared using major ions obtained from DOW. Thus, this study primarily addresses whether these major ions influence the antiliver fibrosis effect of CM.

## 2. Results

### 2.1. Effect of DOW on the Cordycepin and Adenosine Production of CM

Previous studies have reported that DOW can promote the generation of functional metabolites in microorganisms [14]. The results of the present study also show that DCM considerably produced higher contents of cordycepin and adenosine by 2.02 times and 2.31 times than UCM, respectively. To investigate whether the major ions in DOW induce the increase in the content of functional components, SCM was produced using SW as the culture water. Table 1 shows that SCM exhibits higher increase in the cordycepin and adenosine content by 1.62 times and 1.76 times compared with UCM, respectively. However, it is less effective than DCM is. These results indicate that DCM has the highest cordycepin and adenosine contents.

### 2.2. Effect of the Individual Metal Waters on the Production of Monascus-Fermented Pigments

Table 2 shows that eight weeks after the administration of intraperitoneal TAA injection, the male BALB/c mice demonstrated a significantly lower body weight compared with the mice in the NOR group (*p* < 0.001). The liver weight and the ratio of liver weight to body weight of the injected mice were considerably higher than those of the mice in the NOR group (*p* < 0.001). After the mice in both groups were fed the test substances, their liver weight and the ratio of liver weight to body weight decreased, and body weight increased.

### 2.3. Effect on Serum AST, ALT, ALP, and TBIL

Serum aspartate aminotransferase (AST) and alanine aminotransferase (ALT) are vital indicators typically used to test liver function. Alkaline phosphatase (ALP) and total bilirubin (TBIL) are used to test hepatobiliary function [15]. In Table 3, the AST and ALT activities were considerably higher in the TAA group than they were in the NOR group (*p* < 0.001). This indicates that the livers of the mice in the TAA group were severely injured. After the fermented material was administered, the AST activity declined significantly in both groups. The ALT activities in the SL, DCM-H, AS, and CC groups were significantly lower than that in the TAA group (*p* < 0.05). In addition, ALP and TBIL increased substantially in the TAA group compared with the NOR group (*p* < 0.001). Feeding the mice DCM effectively reduced the ALP and TBIL content (*p* < 0.01).

### 2.4. Effect on the Liver HE Stain and Collagen Stain

**Figure 1:**
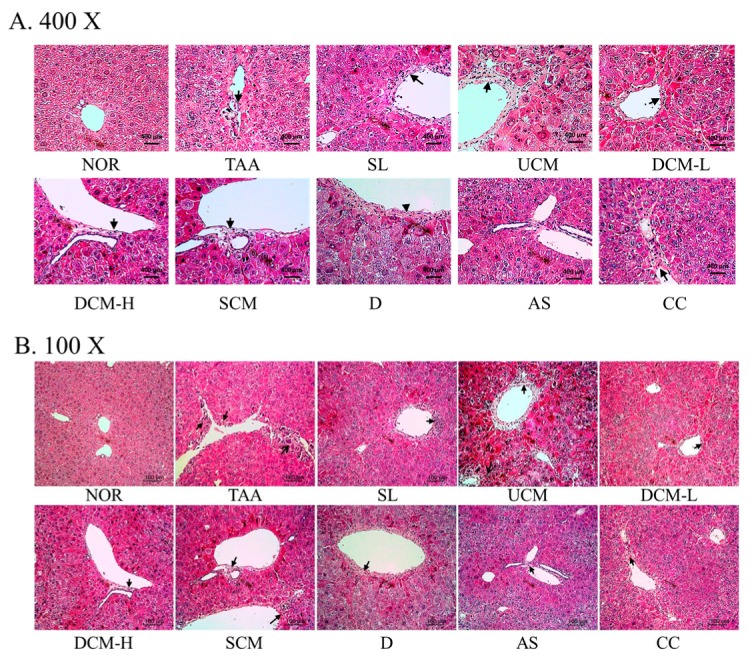
The effects of *C. militaris*-fermented product cultured with various waters on pathological changes in the TAA-induced liver fibrosis mice. (**A**) 400×; (**B**) 100×. NOR: normal group, TAA: TAA-induced mice (TAA 100 mg/kg/ip, three times per week), SL: TAA-induced mice fed silymarin (75 mg/kg/day), UCM: TAA-induced mice fed a one-fold dose of UCM (0.603 g/kg/day), DCM-L: TAA-induced mice fed a one-fold dose of DCM (0.603 g/kg/day), DCM-H: TAA-induced mice fed a three-fold dose of DCM (1.809 g/kg/day), SCM: TAA-induced mice fed a one-fold dose of SCM (0.603 g/kg/day), DOW: TAA-induced mice fed with DOW (0.603 mL/kg/day), AS: TAA-induced mice fed with adenosine (0.329 mg/kg/day), CC: TAA-induced mice fed with cordycepin (0.615 mg/kg/day).

We conducted hematoxylin and eosin staining on liver sections to evaluate the damage to liver tissue. In Figure 1, the results of the stained section obtained from the NOR group indicated intact liver lobules. The hepatic plates were radially arranged. After the TAA injection, the structure of the liver lobules in the TAA group changed and the hepatic plate arrangements were disrupted. Substantial amounts of inflammatory microphages were concentrated around the blood vessels, presenting a severe inflammatory response. Some of the hepatocytes were undergoing apoptosis. The liver lobules of the mice in the DCM-L and DCM-H groups sustained considerably moderate damages compared with those of the mice in the TAA group; in these lobules, markedly fewer inflammatory microphages surrounded the blood vessels, and no hepatocytes presented with apoptosis. Furthermore, although the structures of the liver lobules in the SCM, AS, and CC groups were not intact, and the amounts of inflammatory microphages were clearly reduced. The reduction in the amounts of inflammatory microphages in the SL and D was less than that in the DCM-L group. In CM fermented with UPW (UCM), the liver tissue section results indicated that substantial amounts of inflammatory microphages accumulated around the blood vessels, and the hepatocytes were inclined to undergo apoptosis. No clear recovery effects in response to the damage caused by TAA were exhibited.

**Figure 2:**
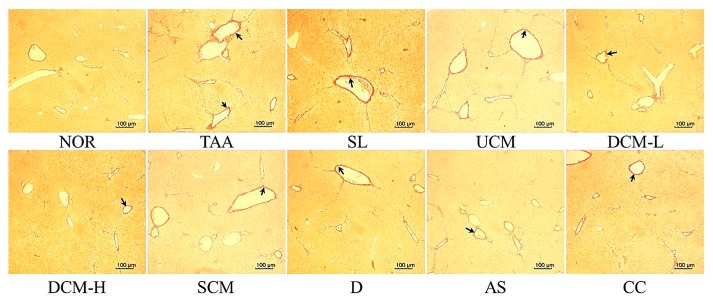
The effects of *C. militaris*-fermented product cultured with various waters on collagen accumulation in the TAA-induced liver fibrosis mice. NOR: normal group, TAA: TAA-induced mice (TAA 100 mg/kg/ip, three times per week), SL: TAA-induced mice fed silymarin (75 mg/kg/day), UCM: TAA-induced mice fed a one-fold dose of UCM (0.603 g/kg/day), DCM-L: TAA-induced mice fed a one-fold dose of DCM (0.603 g/kg/day), DCM-H: TAA-induced mice fed a three-fold dose of DCM (1.809 g/kg/day), SCM: TAA-induced mice fed 1-fold dose of SCM (0.603 g/kg/day), DOW: TAA-induced mice fed with DOW (0.603 mL/kg/day), AS: TAA-induced mice fed with adenosine (0.329 mg/kg/day), CC: TAA-induced mice fed with cordycepin (0.615 mg/kg/day).

Figure 2 shows that the liver tissue of the NOR group demonstrated virtually no accumulation of collagen fiber. After the TAA injection, substantial amounts of collagen accumulated around the blood vessels, and fibrous substances were produced in the TAA group. The collagen fiber content in the liver tissue of the DCM-L, DCM-H, and CC groups was clearly lower than that in the TAA group. Although the SL, SCM, D, and AS groups exhibited reductions in the collagen fiber content, these reductions were lower than those observed in the DCM-L group. No substantial difference in the collagen fiber content was observed between the UCM and the TAA groups.

### 2.5. Effect on the Liver Proinflammatory Factors and PPAR-γ Protein Expression

In chronic liver disease, the inflammatory response is often closely associated with liver fibrosis [16,17]. Inflammatory cytokines such as TNF-α, IL-6, and IL-1β are the main factors controlling the inflammatory response that causes liver damage and liver fibrosis [17,18].

**Figure 3:**
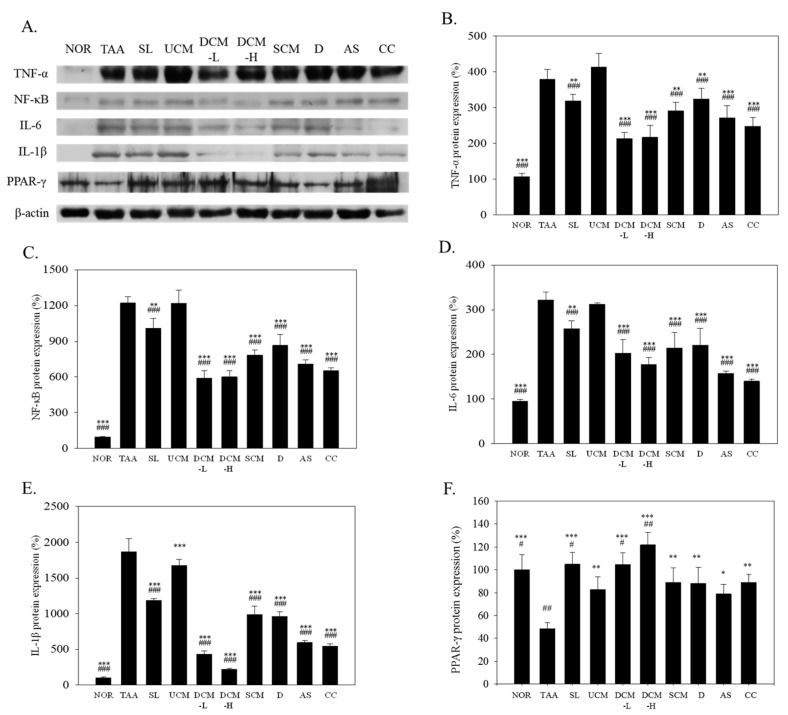
The effect of *C. militaris*-fermented product cultured with various waters on haptatic TNF-α, NF-κB, IL-6, IL-1β, and PPARγ protein expression in the TAA-induced liver fibrosis mice. Target protein expressions were visualized using immunoblotting (**A**) and quantified using gel analysis software (**B**–**E**). NOR: normal group, TAA: TAA-induced mice (TAA 100 mg/kg/i.p., three times per week), SL: TAA-induced mice fed silymarin (75 mg/kg/day), UCM: TAA-induced mice fed a one-fold dose of UCM (0.603 g/kg/day), DCM-L: TAA-induced mice fed a one-fold dose of DCM (0.603 g/kg/day), DCM-H: TAA-induced mice fed a three-fold dose of DCM (1.809 g/kg/day), SCM: TAA-induced mice fed a one-fold dose of SCM (0.603 g/kg/day), DOW: TAA-induced mice fed with DOW (0.603 mL/kg/day), AS: TAA-induced mice fed with adenosine (0.329 mg/kg/day), CC: TAA-induced mice fed with cordycepin (0.615 mg/kg/day). * *p* < 0.05, ** *p* < 0.01, *** *p* < 0.001 vs. TAA group. ^#^ *p* < 0.05, ^##^ *p* < 0.01, ^###^ *p* < 0.001 vs. UCM group.

TNF-α can prompt the activation of HSCs in rats, causing liver fibrosis [19]. In Figure 3A,B, the TNF-α levels increased by 354% (*p* < 0.001) in the TAA group versus the NOR group. After the same doses of the CM-fermented product were fed to the mice, the DCM-L, SCM, and UCM groups exhibited dissimilar reductions in the TNF-α levels. The TNF-α level reductions in the DCM-L and SCM groups were 44% (*p* < 0.001) and 23% (*p* < 0.01), respectively. No increase in the TNF-α level was exhibited in the UCM group (*p* > 0.05). DCM is rich in DOW, adenosine, and cordycepin, all of which inhibit the expression of TNF-α. Compared with that in the TAA group, the TNF-α levels in the D, AS, and CC groups were reduced by 16% (*p* < 0.01), 28% (*p* < 0.001), and 35% (*p* < 0.001), respectively.

Multiple in vitro tests have shown that the activation of HSCs is associated with increases in NF-κB activity [20]. Figure 3A,C illustrates the effects of the test substances on the NF-κB protein levels in liver tissue. The NF-κB levels of the TAA group were higher than those of the NOR group (*p* < 0.001). Compared with that in the TAA group, the NF-κB levels in the DCM-L, DCM-H, and SCM groups were reduced by 52% (*p* < 0.001), 51% (*p* < 0.001), and 36% (*p* < 0.001), respectively. In the D, AS, and CC groups, these levels were reduced by 29% (*p* < 0.001), 42% (*p* < 0.001), and 46% (*p* < 0.001), respectively. They dropped by only 17% (*p* < 0.01) in the SL group, whereas no reduction (*p* > 0.05) was exhibited in the UCM group. These results show that DCM reduced NF-κB levels more significantly than UCM did possibly because DCM contains DOW, adenosine, and cordycepin, all of which inhibit NF-κB levels.

Figure 3A,D shows the observed IL-6 protein levels in liver tissue. The IL-6 protein levels were increased (*p* < 0.001) in the TAA group compared with those in the NOR group. Compared with that in the TAA group, the IL-6 levels in the DCM-L, DCM-H, SCM, D, AS, and CC groups were reduced by 37% (*p* < 0.001), 45% (*p* < 0.001), 33% (*p* < 0.001), 31% (*p* < 0.001), 51% (*p* < 0.001), and 57% (*p* < 0.001), respectively. The levels of this protein dropped by 20% (*p* < 0.01) in the SL group. UCM did not suppress the IL-6 levels induced by TAA (*p* > 0.05).

TAA also induced a significant increase in the IL-1β levels (Figure 3A,E). This is because TAA damages the liver cells after entering the body, leading to the activation of the Kupffer cells and generation of inflammatory cytokines. DCM-L and SCM significantly inhibited the IL-1β levels by 77% (*p* < 0.001) and 47% (*p* < 0.001), respectively. DOW, adenosine, and cordycepin also demonstrated clear inhibitory effects. The inhibitory effect of UCM on the IL-1β levels was relatively weak, reducing the IL-1β levels by only 10%.

Previous study reported that PPAR-γ exhibits excellent anti-inflammatory capabilities [21]. In addition, increases in PPAR-γ levels can inhibit the activation of HSCs [22]. Synthesized PPAR ligands, such as thiazolidinediones, can inhibit the activation of HSCs and reduce the accumulation of collagen proteins [23]. In Figure 3A,F, compared with those in the NOR group, the PPAR-γ protein levels in the TAA group dropped by 48% (*p* < 0.001). After the administration of the test substances, PPAR-γ protein levels in the UCM, DCM-L, DCM-H, SCM, D, AS, and CC groups rebounded by 71% (*p* < 0.01), 116% (*p* < 0.001), 152% (*p* < 0.001), 84% (*p* < 0.01), 83% (*p* < 0.01), 63% (*p* < 0.05), and 84% (*p* < 0.01), respectively, compared with those in the TAA group. The SL group rebounded by 117%. Among the fermented products, DCM-H exhibited more favorable rebound effects than SCM and D did. This indicates that DOW enables CM to prevent liver fibrosis.

### 2.6. Effect on the Liver Fibrosis-Related Factors Expression

**Figure 4:**
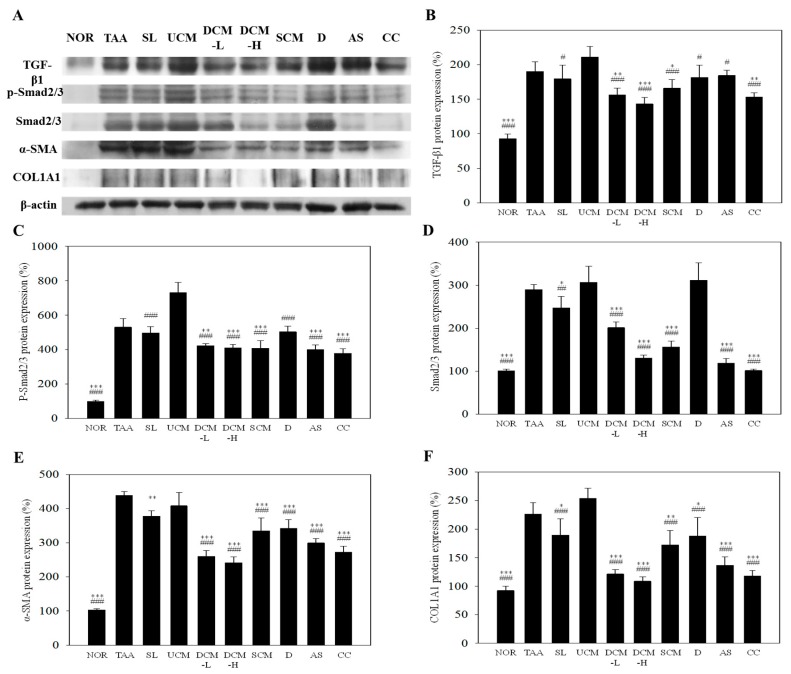
The effect of *C. militaris*-fermented product cultured with various waters on haptatic TGF-β1, p-Smad2/3, Smad2/3, α-SMA, and COL1A1 protein expression in the TAA-induced liver fibrosis mice. Target protein expressions were visualized using immunoblotting (**A**) and quantified using gel analysis software (**B**–**E**). NOR: normal group, TAA: TAA-induced mice (TAA 100 mg/kg/i.p., three times per week), SL: TAA-induced mice fed silymarin (75 mg/kg/day), UCM: TAA-induced mice fed a one-fold dose of UCM (0.603 g/kg/day), DCM-L: TAA-induced mice fed a one-fold dose of DCM (0.603 g/kg/day), DCM-H: TAA-induced mice fed a three-fold dose of DCM (1.809 g/kg/day), SCM: TAA-induced mice fed 1-fold dose of SCM (0.603 g/kg/day), DOW: TAA-induced mice fed with DOW (0.603 mL/kg/day), AS: TAA-induced mice fed with adenosine (0.329 mg/kg/day), CC: TAA-induced mice fed with cordycepin (0.615 mg/kg/day). * *p* < 0.05, ** *p* < 0.01, *** *p* < 0.001 vs. TAA group. ^#^ *p* < 0.05, ^##^ *p* < 0.01, ^###^ *p* < 0.001 vs. UCM group.

TGF-β1 catalyzes the conversion of HSCs into myofibroblast-like cells, stimulating the generation of ECM and inhibiting its degradation [4]. The presence of TGF-β1 products or its signaling pathways are considered anti-inflammatory and potential antifibrotic indicators [4]. Figure 4A,B illustrates the effects of the test substances on the TGF-β1 protein levels in liver tissue. TAA induced the TGF-β1 protein levels to increase by 2.09 times (*p* < 0.001 vs. NOR). Compared with that in the TAA group, the TGF-β1 protein levels in the DCM-H and CC groups were significantly reduced by 19% (*p* < 0.05) and 21% (*p* < 0.05), respectively. However, no reductions were observed in the SL, UCM, DCM-L, SCM, D, and AS groups. Therefore, cordycepin is the active component reducing the TGF-β1 protein levels in DCM.

Smad2/3 is a major factor in TGF-β1 signaling pathways. Inhibiting the phosphorylation and its expression in Smad2/3 also indirectly leads to downstream signaling inhibition [4] preventing the activation of HSCs and reducing liver fibrosis. As depicted in Figure 4A,C, the p-Smad2/3 protein levels increased significantly in the TAA group compared with the NOR group. UCM could not reduce the p-Smad2/3 level induced by TAA. DCM and its main components adenosine (AS group) and cordycepin (CC group) were also only slightly effective at inhibiting the expression of p-Smad 2/3, but they exhibited clear inhibitory effects on the Smad2/3 levels. Compared with that in the TAA group, the Smad2/3 levels in the DCM-L, DCM-H, SCM, AS, and CC groups were reduced by 31% (*p* < 0.001), 55% (*p* < 0.001), 46% (*p* < 0.001), 59% (*p* < 0.001), and 65% (*p* < 0.001), respectively. Although UCM is also a product of CM fermentation, it was ineffective in reducing the Smad2/3 levels (*p* > 0.05). This indicates that adenosine and cordycepin primarily reduce p-Smad2/3 levels by inhibiting Smad2/3 expression rather than directly inhibiting its phosphorylation.

α-SMA levels can be considered as indicators of the existence of activated HSCs. Figure 4A,D shows the effects of the test substances on the α-SMA protein levels in liver tissue. Compared with that in the NOR group, the α-SMA protein levels in the TAA group increased by 4.25 times (*p* < 0.001). After the application of the test substances, the α-SMA protein levels in the DCM-L, DCM-H, SCM, D, AS, and CC groups dropped by 41% (*p* < 0.001), 45% (*p* < 0.001), 23% (*p* < 0.001), 22% (*p* < 0.001), 32% (*p* < 0.001), and 38% (*p* < 0.001), respectively, compared with that in the TAA group. The protein levels in the SL group dropped by 14% (*p* < 0.01). No significant reductions were observed in the UCM group (*p* > 0.05). DCM-H existing in the fermentation product exhibited a significantly more effective inhibitory effect compared with SCM (*p* < 0.01) and D (*p* < 0.001). These results show that CM fermented with DOW is effective at inhibiting the activation of HSCs and that DOW facilitates enhancing the liver protection capabilities of CM.

Collagen type I, the major component of ECM, forms a characteristic triple-helix structure composed of two COL1A1 chains and one COL1A2 chain [24]. The synthesis of collagen type I polypeptides is regulated by the TGF-β_1_ activation protein pathway and the Smad signaling pathway [25]. In the results, TAA significantly induced COL1A1 protein levels (*p* < 0.001 vs. NOR) in liver. DCM-L and DCM-H can inhibit the formation of COL1A1 by 47% (*p* < 0.001) and 51% (*p* < 0.001) as compared with TAA group, respectively. UCM was the default to inhibit the formation of COL1A1 toward the development of liver fibrosis (*p* > 0.05). Adenosine (AS group) and cordycepin (CC group) can be the functional compounds due to the significant inhibition by 39% (*p* < 0.001) and 48% (*p* < 0.001) as compared with TAA group, respectively.

## 3. Discussion

Previous study reported that TAA demonstrates more pronounced stimulating effects on the production of regenerative nodules and fibrosis in the liver compared with CCl_4_ [26]. The pathological changes in liver injuries induced in rats and mice with TAA are similar to those of human liver injuries [27]. Upon injected into the body, TAA metabolizes, producing TASO_2_. Subsequently, TASO_2_ damages the liver cells, stimulating the Kupffer cells and liver cells to secrete proinflammatory cytokines, which cause an inflammatory response. The inflammatory response stimulates the activation of HSCs. Activated HSCs secrete even more cytokines, continuing the inflammatory response. This subjects the HSCs to a state of continual activation, triggering fibrosis and cell proliferation [1,2,3]. Among the cytokines produced by activated HSCs, TGF-β1 plays a pivotal role in regulating liver fibrosis. TGF-β1 can promote the proliferation of activated HSCs and secretion of ECM and inhibit their decomposition [4,5]. TGF-β1 also promotes the activation of HSCs and the expression of collagen type I through the TGF-β1 signaling pathway [25,28]. Increases in PPAR-γ levels are inversely correlated with the number of activated HSCs [23].

Previous studies have determined that CM has powerful protective effects against alcohol-induced damage [29]. However, research has yet to prove that the protective effects of CM on the liver can inhibit liver fibrosis. Therefore, increasing the functional components of CM may facilitate the process of improving its liver protection capabilities. In the present study, we used DOW to stimulate the generation of considerable amounts of cordycepin and adenosine in CM. The DOW that accumulates in the cordyceps solid matrix during the fermentation process may, thus, exhibit protective effects on the liver. These compound components may generate synergies that can increase the liver protection capabilities of DCM-fermented products. Our results showed that UCM improved liver function. However, this improvement was considerably lower than that of CM fermented with DOW. The liver function analysis results did not reveal a difference between the two. However, liver histology and liver collagen staining results showed that the ameliorative effects of DCM were considerably stronger than those of UCM. DOW, adenosine, and cordycepin also improved liver function and prevented liver injury. Although these three substances did not reduce the serum AST activity as much as DCM did, they induced substantial reductions compared with those in the TAA group (*p* < 0.001). Therefore, DCM may be more effective in preventing liver fibrosis because of the accumulation of DOW and because it is rich in adenosine and cordycepin, both of which are increased by DOW.

Studies have shown that DOW may have numerous health benefits because it is rich in minerals and other nutrient salts [14]. In our previous studies, Red mold dioscorea fermented with DOW can significantly perform higher hypolipidaemic effect than those of red mold dioscorea fermented with water filtered through reverse osmosis (RO water) [8]. In addition, Red mold dioscorea fermented with DOW exhibits stronger anti-obesity effect compared with red mold dioscorea fermented with UPW [10]. DOW has also been used to enhance the capability of *A. camphorata* to prevent liver fibrosis [9]. In summary, the literature and the results of the present study show that DOW can promote the health benefits of fungi.

Liver fibrosis is often accompanied by inflammatory responses. Cytokines secreted by Kupffer cells, such as IL-1, IL-6, and TNF-α, activate neutrophils and induce them to reach the inflammation sites in the liver [30]. Neutrophils can indirectly promote the synthesis of collagen fiber in HSCs [3]. TNF-α can induce the activation of HSCs, leading to liver fibrosis in rats [19]. Numerous in vitro tests have also shown that the activation of HSCs is associated with increased NF-κB activity [20]. The inflammatory response is a major risk factor for liver fibrosis. In this study, DCM and its functional components (i.e., adenosine and cordycepin) can considerably inhibit IL-6, IL-1β, TNF-α, and NF-κB protein levels. However, UCM group had no clear anti-inflammatory capabilities. This shows that DCM had excellent anti-inflammatory effects and that these effects are mainly attributable to its greater adenosine and cordycepin contents. Adenosine and cordycepin exhibited similar effects to that of DCM in reducing the level of inflammatory cytokines.

A previous study indicated that PPAR-γ exhibits excellent anti-inflammatory capabilities [21]. Increases in PPAR-γ levels can inhibit the activation of HSCs [22]. Another key indicator for blocking liver fibrosis is the increased expression of PPAR-γ, which inhibits the activation and proliferation of HSCs [23]. DCM increased the PPAR-γ protein levels and its effect was considerably stronger than that of the UCM. In addition, adenosine, cordycepin, and DOW increased PPAR-γ protein levels. Therefore, DCM is highly capable of increasing PPAR-γ protein levels because of its rich adenosine, cordycepin, and DOW contents.

Among fibrosis-related factors, TGF-β1 can promote the conversion of HSCs into myofibroblast-like cells, thereby stimulating the generation of ECM and inhibiting its degradation [4]. TGF-β1 can also indirectly regulate the proliferation of HSCs [5]. Therefore, the presence of TGF-β1 or its signaling pathways are considered anti-inflammatory or potential antifibrosis indicators. TGF-β1 activates HSCs primarily through TGF-β1/Smad signaling pathways, causing liver fibrosis [28]. HSC activation initiates the expression of substantial amounts of α-SMA in smooth muscle. Furthermore, collagen type I, the main component of ECM in liver fibrosis, is also regulated by the TGF-β_1_ activation protein pathway and the Smad signaling pathway [25]. The results of the present study showed that DCM considerably reduced TGF-β1, Smad2/3, α-SMA, and COL1A1 protein levels. However, such reduction was not observed in the UCM group. This indicates that, despite UCM and DCM both being the products of CM fermentation, DCM contains key components lacking in UCM (or has only a limited amount). According to our results, although adenosine does not inhibit TGF-β1 levels, it can inhibit Smad2/3 levels and phosphorylation and reduce α-SMA and COL1A1 protein levels. The other functional component, cordycepin, can reduce TGF-β1, Smad2/3, α-SMA, and COL1A1 protein levels. Previous study reported that cordycepin can interfere with the TGF-β1 signaling pathway by inhibiting Smad protein levels and phosphorylation, thereby preventing the activation of HSCs [13]. Although DOW cannot reduce TAA-induced TGF-β1, Smad2/3, and p-Smad2/3 protein levels, it can considerably reduce α-SMA and COL1A1 protein levels. We infer that this is caused by the DOW-induced reduction of the proinflammatory factor expressions, thereby inhibiting the activation of HSCs by TNF-α and NF-κB and further terminating the expression of α-SMA and COL1A1 protein. According to these results, adenosine, cordycepin, and DOW can ultimately reduce the expression of α-SMA. Therefore, we infer that these three substances are the critical components enabling DCM to improve liver fibrosis more than UCM does.

The aforementioned results show that DOW mitigates TAA-induced liver fibrosis. DOW is also rich in nutrients and trace elements such as magnesium and selenium, which has been reported to be the reason that DOW exhibits numerous health benefits [14]. A previous study reported that magnesium chenoursodeoxycholic acid can reduce the accumulation of collagen fibers and the inflammatory response triggered by CCl_4_ by reducing TNF-α, IL-6, and COX-2 protein levels [31]. In addition, in models of dimethylnitrosamine-induced liver cirrhosis in rats, N-acetylcysteine-magnesium can substantially reduce the inflammatory response and fibrosis in the liver and has hepatoprotective effects [32]. DOW also contains selenium, which is a key cofactor of glutathione peroxidase in the antioxidative system. The results of the present study indicated that DOW inhibited α-SMA protein levels (*p* < 0.001), thereby reducing the occurrence of liver fibrosis. According to the mentioned studies, we infer that this effect is caused by the magnesium and selenium in DOW. Microorganisms can absorb these inorganic minerals and convert them into organic minerals, increasing bioavailability and enhancing their effects [33].

In the present study, we used ion synthetic water to culture CM through fermentation. SW and DOW contain equal amounts of major ions (i.e., Mg, Na, Ca, K, and Fe). The purpose was to determine whether these major ions stimulated DOW to enhance the liver fibrosis preventive ability of CM. The results revealed that SCM, among other indicators, also improved liver fibrosis. Specifically, SCM can generate the equal effects as DCM does in inhibiting TGF-β1 and Smad2/3 expression. However, SCM exhibited weaker inhibitory effects on IL-1β, α-SMA, and COL1A1 levels, as well as weaker stimulating effects on the PPAR-γ levels compared with DCM. This indicates that the major ions in SW functioned similarly as those in DOW, preventing liver fibrosis. DOW may, nonetheless, contain numerous other ions or functional components that grant optimal anti-liver fibrosis effects to CM.

In summary, the presented results show that because DOW is added to the fermentation process, DCM contains substantially increased amount of adenosine and cordycepin, and DOW is accumulated in the fermented product. DCM has inhibitory effects against the expression of fibrosis markers, fibrosis-related factors, and the amount of collagen generated during the liver fibrosis process; these effects are considerably greater than the ameliorative effects exerted by UCM. This study confirms that DOW can inhibit the expression of the TAA-induced proinflammatory factors and increase PPAR-γ levels. The increased adenosine and cordycepin promoted by DOW also possess substantial anti-inflammatory effects and can inhibit the expression of fibrosis-related factors and promote PPAR-γ levels. Therefore, because DCM is rich in DOW and functional components, it produces higher antiliver fibrosis effect. DCM can achieve the following functions: (1) it markedly reduces the levels of the proinflammatory cytokine IL-6 and TNF-α and inhibits NF-κB protein levels, reducing the generation of inflammatory cytokines; (2) it substantially increases PPAR-γ levels, which resists proinflammatory factors; and (3) it considerably reduces the levels of TGF-β1, a key profibrogenic factor. It also inhibits Smad2/3 protein levels and phosphorylation in the signaling pathway.

## 4. Experimental Section

### 4.1. Chemicals

Potato dextrose agar (PDA) and potato dextrose broth (PDB) were purchased from Difco Co. (Detroit, MI, USA). Ethanol (95%) was purchased from Taiwan Tobacco and Liquor Co. (Taipei, Taiwan). Silymarin and TAA were purchased from Sigma Chemical Co. (St. Louis, MO, USA). Folin-Ciocalteau agent and gallic acid were purchased from Panreac Quimina S.A. (Barcelona, Spain). Phenol, sulfuric acid, and sodium carbonate were purchased from Merck Co. (Darmstadt, Germany). Anti-PPARγ polyclonal antibody (07-466), anti-TNF-α polyclonal antibody (AB2148P), anti-α-SMA monoclonal antibody (04-1094), and anti-collagen type I polyclonal antibody (AB765P) were purchased from Merck KGaA. (Millipore) (Darmstadt, Germany). Anti-IL-6 polyclonal antibody (sc-1265), anti-IL-1β polyclonal antibody (sc-7884), anti-NF-κB p65 monoclonal antibody (sc-8008), and anti-TGF-β1 (v) polyclonal antibody (sc-146) were purchased from Santa Cruz Biotechnology, Inc. (Dallas, TX, USA). Anti-Smad7 polyclonal antibody (ARP32008_P050) was purchased from Aviva Systems Biology Co. (San Diego, CA, USA). Anti-β-actin monoclonal antibody (MA5-15739) was purchased from Thermo Fisher Scientific Inc. (Rockford, IL, USA). Anti-Smad2/3 monoclonal antibody (8685) and anti-p-smad2/3 monoclonal antibody (8828) were purchased from Cell Signaling Technology, Inc. (Danvers, MA, USA).

### 4.2. The Source of DOW

The concentrated DOW provided from the Eastern Taiwan Deep Sea Water Innovation and Research Center (Taitung, Taiwan) was pumped from a depth of 670 m in the Pacific Ocean near the Eastern Taiwan and processed though the electrodeionization and vacuum concentration. According to our previous study, DOW including 20.65 mg/L Mg^2+^ was defined as one-fold DOW. In this study, 15-fold DOW (including 309.75 mg/L Mg^2+^) was prepared by the dilution of concentrated DOW (including 43,400 mg/L Mg^2+^) with UPW. The concentrations of the trace elements and minerals in 15-fold DOW included 309.75 mg/L Mg, 163.5 mg/L Na, 66 mg/L K, 2.565 mg/L Ca, 4.275 µg/L Fe, 0.075 mg/L Zn, 0.578 mg/L nitrate, 0.030 mg/L Se, 324.5 mg/L sulfate, 8.4 mg/L H_2_SiO_3_, 1.65 mg/L phosphate, and 0.949 g/L chloride. SW was prepared by mixing the standard solution of 309.75 mg/L Mg, 2.565 mg/L Ca, 163.5 mg/L Na, 66 mg/L K, 4.275 µg/L Fe, and 0.075 mg/L Zn ions with equal concentrations to that in 15-fold DOW.

### 4.3. Microorganism and Seed Cultures

*Cordyceps militaris* BCRC 32,219 was purchased from the Bioresource Collection and Research Center (Hsinchu, Taiwan). *C. militaris* was maintained on yeast and mold (YM) agar at 24 °C and transferred to fresh medium for 10 days intervals. Seed cultures were prepared by transferring a loopful of colony from the YM agar slant into a 500-mL Hinton flask containing 100 mL medium (3 g/L yeast extract, 5 g/L malt extract, 10 g/L peptone, 3 g/L dextrose). The cultures were incubated at 28 °C and 100 rpm for five days. After that, inoculum sizes of 5% was transferred to solid cultured substrate.

### 4.4. Solid Fermentation of C. militaris in DOW, SW, or UPW

Thirty grams of oat substrate was soaked in 30 mL 15-fold concentrated DOW, SW, or UPW, and then was autoclaved for 20 min at 121 °C in a 500-mL glass bottle. After being cooled, the substrate was inoculated with a 10% (*v*/*w*) seed culture medium. The inoculated substrate was cultured at 24 °C for 20 days in a dark incubator. After dark culture, *C. militaris* was then cultured at 14 °C under a 12 h light: 12 h dark cycle (light on at 6:00) for 60 days. After fermentation, the crushed and dried product was used for the experiments

### 4.5. Cordycepin and Adenosine Analysis

The powder of *C. militaris*-fermented product (0.1 g) was extracted, respectively, with 1 mL of methanol at 50 °C for 1 h. The extracts (10%, *w*/*v*) were further filtered with a 0.45 μm pore size filter and analyzed by HPLC (Model L-2130, Hitachi Co., Tokyo, Japan) on a C_18_ column (25 cm × 4.6 mm i.d., 5 μm, Phenomenex^®^ LUNA) using the gradient elution.. HPLC was performed according to the method described previously (Yu et al., 2007) in triplicate. Cordycepin and adenosine were separated by gradient elution using the mobile phase with the composition of water-methanol (95.0/5.0 to 58.4/41.6 in 20 min, *v*/*v*). The flow rate was set at 0.8 mL/min. Cordycepin and adenosine were detected using a photodiode array detector (Model L-2455 DAD, Hitachi Co.) set at 260 nm and full wavelength.

### 4.6. Animal Experiments

Male BALB/c mice at six weeks of age were kept in a temperature controlled room (23 °C) under a 12L:12D cycle (light on at 6:00) and were given free access to food and water. In the experiment, 80 mice were randomly divided to 10 groups. The animal model of liver fibrosis was induced by TAA injection according to the previous study [34]. For eight weeks, two groups of the mice were intraperitoneally (i.p.) injected with vehicle solution (NOR group) or TAA (100 mg/kg bw) (TAA group) three times per week as well as daily orally administrated with ROW. The other groups were i.p. injected with TAA (100 mg/kg bw) three times per week, as well as orally administrated daily with silymarin (75 mg/kg/day) (SL group), one-fold dosage of UCM (603 mg/kg/day) (UCM-L group), one-fold dosage (603 mg/kg/day) of CM fermented using 15-fold concentrated DOW (DCM-L group), three-fold dosage (1809 mg/kg/day) of CM fermented using 15-fold concentrated DOW (DCM-H group), one-fold dosage (603 mg/kg/day) of CM fermented using synthetic water (SCM-L group), 15-fold concentrated DOW (603 µL/kg/day) (DOW group), adenosine (0.329 mg/kg/day) (AS group) with equal adenosine concentration to DCM-L group, and cordycepin (0.615 mg/kg/day) (CC group) with equal cordycepin concentration to DCM-L group. The dosage of CM is calculated in accordance with Boyd’s Formula of Body Surface Area as recommended by the FDA (Food and Drug Administration) [35,36]. Feeding mice with CM at a one-fold dosage (603 mg/kg bw) per day corresponds to daily supplementing with 2.942 g of dried CM powder for an adult. Each sample was orally administrated to the mice by stomach tube in each group.

After 10 weeks, the mice were deprived of food for 16 h before being scarified by CO_2_ asphyxiation. Blood samples were collected from the posterior vena cava and centrifuged at 700× *g* for 10 min; the serum was stored at −20 °C until analyzed. Liver tissues were removed and weighed. Portions of the biggest leaf of liver tissue were immersed in 10% formaldehyde for histological inspection. Half of other portion was ground in ice-cold phosphate buffer saline (PBS) and then centrifuged (8000× *g*, 15 min). The other tissue (100 mg) was homogenated in 1.0 mL of lysis buffer (1% Triton X-100, 20 mM Tris, pH 7.5, 100 mM NaCl, 40 mM NaF, 0.2% SDS, 0.5% deoxycholate, 1 mM EDTA, 1 mM EGTA, and 1 mM Na_3_VO_4_) and brief sonication (10 s). The homogenate was centrifuged at 100,000× *g* for 30 min and the supernatant was used for immunoblotting assay. The experiment was reviewed and approved by the Animal Care and Research Ethics Committee of the National Taitung University.

### 4.7. Serum Biochemical and Fibrosis Markers Analyses

Aspartate aminotransferase (AST), alanine aminotransferase (ALT) and alkaline phosphatase (ALP) activities, and total bilirubin (TBIL) in serum, were measured using the commercial kits (Randox Laboratories Ltd., Antrim, UK).

### 4.8. Immunoblotting

Protein concentration was determined by bicinchoninic acid (BCA) method. A total of 40 μg of total protein from each sample was applied for Western blot representative of three independent experiments according to the previous studies [37,38]. The samples were separated on 10% SDS-PAGE gels and transferred to polyvinylidene fluoride membranes. After blocking in a BSA solution, blots were incubated with anti-PPARγ polyclonal antibody, anti-TNF-α polyclonal antibody, anti-α-SMA monoclonal antibody, anti-COL1A1 polyclonal antibody, anti-IL-6 polyclonal antibody, anti-IL-1β polyclonal antibody, anti-NF-κB p65 monoclonal antibody, anti-TGF-β1 polyclonal antibody, anti-Smad2/3 monoclonal antibody, or anti-p-smad2/3 monoclonal antibody at room temperature for 1 h. Then, bands were incubated with specific horseradish peroxidase (HRP)-conjugated secondary antibodies at room temperature for 1 h and visualized by enhanced chemiluminescence (ECL) substrate with UVP AutoChemi Image system (UVP Inc., Upland, CA, USA). Protein loading was evaluated by anti-β-actin antibody (1:1000).

### 4.9. Histological Analysis and Collagen Staining

Liver tissue sections were cut at a thickness of 7-μm and mounted on silanized slides (Dako Japan, Tokyo, Japan). The sections were stained with hematoxylin eosin (HE) to observe the histological features of the livers. Collagen staining of liver tissue section was stained using the picro-sirius red solution (0.1% sirius red in saturated picric acid). By this procedure collagen is stained red [39].

### 4.10. Statistics

Data are expressed as means ± standard deviation. Analysis of variance by Duncan’s test and Pearson’s product-moment correlation coefficient test were determined using SPSS version 12.0 software (SPSS Institute, Inc., Chicago, IL, USA). Differences with *p* < 0.05 were considered statistically significant.

## 5. Conclusions

In summary, the presented results show that because DOW is added to the fermentation process, DCM contains substantially increased amount of adenosine and cordycepin, and DOW is accumulated in the fermented product. DCM has inhibitory effects against the expression of fibrosis markers, fibrosis-related factors, and the amount of collagen generated during the liver fibrosis process; these effects are considerably greater than the ameliorative effects exerted by UCM. This study confirms that DOW can inhibit the expression of the TAA-induced proinflammatory factors and increase PPAR-γ levels. The increased adenosine and cordycepin promoted by DOW also possess substantial anti-inflammatory effects and can inhibit the expression of fibrosis-related factors and promote PPAR-γ levels. Therefore, because DCM is rich in DOW and functional components, it produces higher antiliver fibrosis effect. DCM can achieve the following functions: (1) It markedly reduces the levels of the proinflammatory cytokine IL-6 and TNF-α, and inhibits NF-κB protein levels, reducing the generation of inflammatory cytokines; (2) it subs Shek tantially increases PPAR-γ levels, which resists proinflammatory factors; and (3) it considerably reduces the levels of TGF-β1, a key profibrogenic factor. It also inhibits Smad2/3 protein levels and phosphorylation in the signaling pathway.

## Figures and Tables

**Table 1:** Cordycepin and adenosine concentrations of *C. militaris*-fermented oats cultured with various types of water.

Groups	Cordycepin Concentration (mg/g)	Adenosine Concentration (mg/g)
UCM	505.12 ± 16.91 ***	235.18 ± 77.26 ***
DCM	1020.45 ± 76.42	544.30 ± 24.45
SCM	820.60 ± 75.25 *	413.08 ± 10.17 *

UCM, DCM, and SCM were the fermented oats of *C. militaris* cultured with UPW, DOW, and SW, respectively. The concentration of Mg^2+^, Na^+^, K^+^, Ca^2+^, and Fe^2+^ in SW were equal to that in DOW, respectively. Data are presented as mean ± SD (*n* = 3). * *p* < 0.05, *** *p* < 0.001 vs. DCM.

**Table 2:** The effects of *C. militaris*-fermented products cultured with various waters on the body weight gain, liver weight, and liver weight/body weight ratio of the TAA-induced liver fibrosis mice.

Groups	Liver Weight Gain (g)	Body Weight (g)	Liver Weight/Body Weight Ratio (%)
NOR	1.24 ± 0.06 ***^, ###^	29.36 ± 1.17 ***^, ###^	4.51 ± 0.14 ***^, ###^
TAA	2.07 ± 0.09 ^###^	23.34 ± 1.05 ^###^	7.88 ± 0.19 ^###^
SL	1.95 ± 0.07 ***	27.35 ± 1.03 ***	7.15 ± 0.24 ***
UCM	1.91 ± 0.08 ***	27.31 ± 0.46 ***	7.20 ± 0.24 ***
DCM-L	1.90 ± 0.06 ***	27.88 ± 0.59 ***	6.98 ± 0.36 ***
DCM-H	1.93 ± 0.04 ***	27.26 ± 1.15 ***	7.09 ± 0.31 ***
SCM	1.81 ± 0.04 ***^, ##^	25.79 ± 0.55 ***	7.10 ± 0.18 ***
DOW	1.89 ± 0.06 ***	26.96 ± 0.82 ***	7.17 ± 0.15 ***
AS	1.69 ± 0.08 ***^, ###^	25.76 ± 0.93 ***	7.10 ± 0.22 ***
CC	1.75 ± 0.05 ***^, ###^	25.01 ± 0.87 ***	6.95 ± 0.24 ***

NOR: normal group, TAA: TAA-induced mice (TAA 100 mg/kg/ip, three times per week), SL: TAA-induced mice fed silymarin (75 mg/kg/day), UCM: TAA-induced mice fed a one-fold dose of UCM (0.603 g/kg/day), DCM-L: TAA-induced mice fed 1-fold dose of DCM (0.603 g/kg/day), DCM-H: TAA-induced mice fed a three-fold dose of DCM (1.809 g/kg/day), SCM: TAA-induced mice fed a one-fold dose of SCM (0.603 g/kg/day), DOW: TAA-induced mice fed with DOW (0.603 mL/kg/day), AS: TAA-induced mice fed with adenosine (0.329 mg/kg/day), CC: TAA-induced mice fed with cordycepin (0.615 mg/kg/day). Data are presented as mean ±SD (*n* = 8). * *p* < 0.05, ** *p* < 0.01, *** *p* < 0.001 vs. TAA group. ^#^ *p* < 0.05, ^##^ *p* < 0.01, ^###^ *p* < 0.001 vs. UCM group.

**Table 3:** The effects of *C. militaris*-fermented product cultured with various waters on serum AST, ALT, ALP activities, and TBIL contents in the TAA-induced liver fibrosis mice.

Groups	AST (U/L)	ALT (U/L)	ALP(U/L)	TBIL (mg/dL)
NOR	80.63 ± 3.89 ***^, ##^	43.25 ± 2.55 ***^, ###^	97.63 ± 4.93 ***^, ###^	0.043 ± 0.007 ***^, ###^
TAA	124.50 ± 11.12 ^###^	138.75 ± 10.12	202.63 ± 7.48 ^###^	0.109 ± 0.010 ^##^
SL	94.38 ± 4.84 ***	114.75 ± 5.68 *	175.00 ± 16.29 ***	0.091 ± 0.010 ***
UCM	92.25 ± 3.45 ***	126.63 ± 12.23	170.63 ± 8.90 ***	0.094 ± 0.005 **
DCM-L	89.25 ± 5.57 ***^, #^	128.88 ± 10.79	174.63 ± 13.71 ***	0.086 ± 0.009 ***
DCM-H	90.75 ± 6.92 ***	119.63 ± 7.44 *	171.63 ± 14.09 ***	0.086 ± 0.009 ***
SCM	97.63 ± 9.02 ***	129.88 ± 14.51	176.13 ± 5.30 ***	0.086 ± 0.012 ***
D	96.25 ± 8.97 ***	128.63 ± 13.90	177.13 ± 9.43 ***	0.095 ± 0.009 **
AS	99.00 ± 4.78 ***	125.38 ± 17.73 *	161.75 ± 11.52 ***	0.071 ± 0.011 ***^, ###^
CC	97.13 ± 10.59 ***	106.75 ± 10.50 *^, ##^	176.38 ± 10.01 ***	0.080 ± 0.011 ***^, #^

NOR: normal group, TAA: TAA-induced mice (TAA 100 mg/kg/i.p., three times per week), SL: TAA-induced mice fed silymarin (75 mg/kg/day), UCM: TAA-induced mice fed a one-fold dose of UCM (0.603 g/kg/day), DCM-L: TAA-induced mice fed a one-fold dose of DCM (0.603 g/kg/day), DCM-H: TAA-induced mice fed a three-fold dose of DCM (1.809 g/kg/day), SCM: TAA-induced mice fed a one-fold dose of SCM (0.603 g/kg/day), DOW: TAA-induced mice fed with DOW (0.603 mL/kg/day), AS: TAA-induced mice fed with adenosine (0.329 mg/kg/day), CC: TAA-induced mice fed with cordycepin (0.615 mg/kg/day). Data are presented as mean ± SD (*n* = 8). * *p* < 0.05, ** *p* < 0.01, *** *p* < 0.001 vs. TAA group. ^#^ *p* < 0.05, ^##^ *p* < 0.01, ^###^ *p* < 0.001 vs. UCM group.
